# Supplementary material for: Sociodemographic data and APOE-ε4 augmentation for MRI-based detection of amnestic mild cognitive impairment using deep learning systems
Source: PLoS One. 2020 Sep 25;15(9):e0236868. doi: 10.1371/journal.pone.0236868 (PMC7518632; doi:10.1371/journal.pone.0236868)
Supplement: Attachment — Submitted filename: Response_to_Reviewers.pdf [file pone.0236868.s001.pdf]

Editor

1. Please ensure that your manuscript meets PLOS ONE's style requirements, including those for file naming.

Thank you for the comment. The authors have followed the styling guidelines and made the appropriate changes.

2. Please provide additional details regarding participant consent. In the Methods section, please ensure that you have specified (1) whether consent was informed and (2) what type you obtained (for instance, written or verbal). If your study included minors, state whether you obtained consent from parents or guardians. If the need for consent was waived by the ethics committee, please include this information.

All participants provided written informed consent.

3. "Ethics Statement from IRB of University Hospital Essen, Essen, Germany available dated 2009-10-20 to Prof. Dr. C. Weimar, registration number: 06-3116" a. Please amend your current ethics statement to confirm that your named institutional review board or ethics committee specifically approved this study.

Thank you. The ethics statement has been correctly amended.

4. We note that you have indicated that data from this study are available upon request. a) If there are ethical or legal restrictions on sharing a de-identified data set, please explain them in detail (e.g., data contain potentially identifying or sensitive patient information) and who has imposed them (e.g., an ethics committee). Please also provide contact information for a data access committee, ethics committee, or other institutional body to which data requests may be sent.

De-identified data from this study are available upon request to Department of Neurology, University Hospital Essen, Germany pending approval by the study steering committee. Due to data security reasons (i.e., data contain potentially participant identifying information), the HNR Study does not allow sharing data as public use file. Data requests can be addressed to: recall@uk-essen.de

5. We note that you received funding from a commercial source: Celgene GmbH München, Imatron/GE-Imatron, Janssen, Merck KG, Philips, ResMed Foundation, Roche Diagnostics, Sarstedt AG&Co, Siemens HealthCare Diagnostics and the Volkswagen Foundation. Please provide an amended Competing Interests Statement that explicitly states this commercial funder, along with any other relevant declarations relating to employment, consultancy, patents, products in development, marketed products, etc.

Competing intereset were amended.

6. Please ensure that you refer to Table 9 in your text as, if accepted, production will need this reference to link the reader to the Table.

Table 9 has been correctly referred to.

7. Thank you for including your ethics statement on the submission form:
"Ethics Statement for the use of the HNR study population from IRB of University Hospital Essen, Essen, Germany available dated 2009-10-23 and 2012-06-06 to Prof. Dr. C. Weimar, registration number: 06-3116. All participants provided informed consent.
Details about the Ethics statement of the ADNI study population can be found at: https://adni.loni.usc.edu"

The ethics statement has been amended and added to the Methods section of the manuscript.

8. Thank you for clarifying in your Response to Reviewers that: "All participants provided written informed consent."
a) To help ensure that the wording of your manuscript is suitable for publication, would you please also add this statement at the beginning of the Methods section of your manuscript file.

This statement has been added to "Study Population" of the Methods section of the manuscript.

10. Thank you for providing the following data availability statement:
"De-identified data from this study are available upon request to Dr. Martha Jokisch pending approval by the study steering committee. Due to data security reasons (i.e., data contain potentially participant identifying information), the HNR Study does not allow sharing data as public use file. Data requests can be addressed to: recall@uk-essen.de
Additional Data used in preparation of this article were obtained from the Alzheimer's Disease Neuroimaging Initiative (ADNI) database (https://adni.loni.usc.edu). Details about data access is detailed there."

Before we proceed, please address the following points:
a) We note de-identified data are available upon request to Dr. Jokisch. PLOS policy does not allow authors to be the sole point of contact for data access queries. Please provide a non-author point of contact for fielding de-identified data access queries from future researchers.

We have provided a non-author point for data access queries and added this information to journal editor comments point 4.

b) Please provide any relevant accession codes, data set names, etc a future researcher may need when requesting access to the additional data obtained from the Alzheimer's Disease Neuroimaging Initiative (ADNI) database.

There are no relevant accession codes or data set names.

c) Please also confirm the authors had no special access privileges others would not have to the data obtained from the Alzheimer's Disease Neuroimaging Initiative (ADNI) database.

The authors had no special access privileges others would not have to the data obtained from the Alzheimer's Disease Neuroimaging Initiative (ADNI) database.

Reviewer 1

Introduction section:

1. Page 2, line 11: “The amnestic MCI (aMCI) subtype has a high probability of progressing to AD”. Indeed, AD neuropathology could be present and related to aMCI by the time aMCI is diagnosed. In this case1, aMCI should correspond to first symptomatic presentation of AD. Therefore, I suggest including the term "dementia" to AD instead of only AD.

We have included the term “dementia” accordingly.

2. The authors should explain previous works and knowledge development as a history. I suggest they avoid citation like this one in page 2, line 20: “As shown in [6–9], multi-modal representation achieves higher prediction rates in medical and biomedical annotation tasks...”. Also, in page 2, line 38: “In [17, 18], automatic generated keywords are combined with x-rays to obtain optimized body region classification”. And, in page 2, line 44: “In [22, 23], late fusion methods were applied, where decision values from several classifiers are fused to make the final classification prediction”. The authors frequently cite previous works by the references. I suggest they cite the authors first, as a history of development of knowledge, and, then, they should include the citation number.

The reference style was changed. The authors were first cited by their name and then the citation number.

Methods section:

3. Page 3, line 88: the authors explain that the current study is a sub-study derived from the Heinz Nixdorf Recall (HNR) Study. They relate that the verbal memory, orientation/praxis, information processing speed, executive functions and verbal abilities were covered by the study protocol. Was the aMCI diagnosis based on the results of the short or on more comprehensive neuropsychological assessment?

The aMCI diagnosis was based on a neurological and neuropsychological evaluation. The neuropsychological evaluation was performed by an experienced neuropsychologist using the more comprehensive assessment (see point below).

4. What was considered the gold standard method for diagnosing aMCI?

The gold standard method consisted of a neurological and neuropsychological evaluation. The neurological evaluation was performed by a senior neurologist. The neuropsychological examination was performed by an experienced neuropsychologist. The final decision about aMCI diagnosis was ultimately made by consensus agreement between the examining neurologist and neuropsychologist taking into account the medical history related to cognitive functioning, duration of such symptoms, the history of other medical illnesses and current treatment for each participant. Participants with aMCI had to fulfill the following criteria: Cognitive impairment in the verbal memory domain; presence of subjective cognitive decline; normal functional abilities of daily living and no dementia diagnosis.

We have now added this information to the “Evaluation of cognitive status and aMCI diagnosis” section as follows:

"The final decision about aMCI diagnosis was ultimately made by consensus agreement between the examining neurologist and neuropsychologist taking into account the medical history related to cognitive functioning, duration of such symptoms, the history of other medical illnesses and current treatment for each participant."

5. When was the current study conducted?

The data used for these analyses were assessed from 2006 to 2009.This information has been added to the "Study population" section.

6. Considering that current study is a sub-study derived from the Heinz Nixdorf Recall (HNR) Study, what were the inclusion and exclusion criteria?

Inclusion criteria: *A short cognitive assessment was performed in the HNR study (see Wege et al. 2011, Neuroepidemiology). A random sample of participants (aged 50–80 years) with impaired screening results (two subtests below the age- and education adjusted mean) and age-appropriate screening results were invited to the sub-study.*

Exclusion criteria: *Participants with dementia, severe depression (ADAS depression subscale score >4), Parkinson disease, mental retardation, severe alcohol consumption (for women: >20 g/day; for men: >40 g/day), known brain cancer, severe problems with the German language (foreign persons) and severe sensory impairment leading to invalid cognitive testing were excluded.*

We have added this information to the "Study population" section.

7. Page 3, line 107: the authors explained "Because the MCI due to AD criteria by Albert et al. [42] were not yet published when the sub-study started, the Winblad et al. [43] MCI criteria were used to diagnose aMCI. Therefore, the diagnosis of aMCI was equivalent to the diagnosis of MCI due to AD without biomarker information representing the core clinical criteria as proposed by Albert et al".

We totally agree with the reviewer and have now added this limitation to our "discussion" section as follows:

"It has to be kept in mind that we did not have any biomarker information that is specific for hallmark AD proteinopathies like amyloid beta deposition or phosphorylated tau. Thus, we cannot identify the underlying pathology in our aMCI cases."

8. The authors included sociodemographic characteristics and APOE-ε4 genotyping as study covariates. Why didn´t they include clinical characteristics, especially cardiovascular risk factors, as covariates?

We totally agree with the reviewer, however other clinical characteristics were not added as they were not part of the proof of principle.

9. Page5, line 135: "Table 1 shows the distribution of the sociodemographic data variables age, gender, education, marital status and genetic data variable APOE-ε4 genotype (defined as "clinical data") for aMCI and cognitively unimpaired controls on the applied sub sample". Why did the authors consider genetic data as clinical data? Please, explain it.

Thank you for the comment. To avoid confusion, the authors have changed "clinical data" to "participant data". Participant data is further split into sociodemographic (age, gender, education and marital status) and genetic data (APOE)

10. Page 5, line 142: "The presented work proposes an approach to fuse clinical data with MRI scans, enabling enriched multi-modal image representation". Is it possible to consider sociodemographic characteristics and APOE-ε4 genotype as clinical data?

Thank you for the comment. The authors have removed the misleading sentences.

11. Page 5, line 142: "This is fundamental for image classification and retrieval purposes, and is not limited to computer-aided decision systems for clinical diagnoses, as positive results have been presented for 2D Images in Pelka et al. [21]. The method of encoding clinical data onto 2D slices of an MRI scan is denoted in this work as "Branded". Since current studies have used 3D image-processing, using MRI 2D slices does not constitute a study limitation? Please, explain it better.

The limitations regarding MRI 2D slices vs 3D have been added.

12. Page 6: Table 2 needs a legend with explanations.

Legends have been added to Table 2.

13. I think statistical analysis needs to be more detailed.

Additional analysis has been written. This is also the case for the second dataset (ADNI Phase 1) used for further evaluation.

Discussion section:

14. Discussion is good, based on the results. However, it is important to emphasize the limitations, especially lack of clinical data and of biomarkers of AD.

Thank you for the comment. The authors have removed the misleading sentences.

Conclusion:

15. Page 11, line 325, the authors conclude "Information fusion is obtained by encoding the values of the clinical data variables: gender, marital status, APOE-ε4, age and education as markers and branding these on the MRI scans, prior to training and prediction. I advise that it is important to pay attention to "clinical data variables".

Thank you for the comment. The authors have removed the misleading sentences.

Reviewer 2

Major comments:

Overall, methodologically, I think using the transfer learning on 2D MRI slices, with some kind of augmentation to fit in the RGB channels, and using LSTM to model information across slices are much like very early deep learning works in this medical imaging, when people tried to fit the medical image into successful deep learning models demonstrated elsewhere. I think the field has moved beyond it. This also relates to my point below, where large dataset can make a difference in methodological choice. For the specific study design, the main concern is the low number of subjects, as the authors also realized. We can see the large variance present in Table 3. There are many external large public datasets (ADNI, OASIS, AIBL, …) to test the algorithm. And due to the restriction on the data of this study, future researchers are also not able to compare their results with the result present in this study.

Thank you for the comment and we totally agree that an additional and open-accessible state-of-the-art dataset should be included. Limitations regarding 2D vs 3D models are now mentioned in the manuscript. The authors have further evaluated the proposed approach on the ADNI Phase 1 dataset. Using the same participants data structure and branding process, we reproduce similar predication scores for the classification of controls vs aMCI. This manuscript was submitted prior to the ADNI Data and Publications Committee and received approval regarding Data User Agreement. In this manuscript, ADNI is acknowledged, the data gathering is described in the Methods section, and included to the named authors.

Minor comments:

1. dCNN and DeCafs are not good abbreviations for those well-known concepts.

The abbreviation dCNN and DeCafs have been removed. DeCafs was changed throughout the manuscript to DeCAF.

2. I have heard little usage of the term 'prediction rate', maybe change to more common terms such as prediction accuracy.

Prediction rate was changed to prediction accuracy.

3. The related work section is a bit confusing and unrelated, such as multiple image and text fusion references, the hearing loss paper. Some of the more related references might include:
Spasov, Simeon, et al. "A parameter-efficient deep learning approach to predict conversion from mild cognitive impairment to Alzheimer's disease." Neuroimage 189 (2019): 276-287.
Yang, Jie, et al. "Characterizing Alzheimer's Disease with Image and Genetic Biomarkers using Supervised Topic Models." IEEE journal of biomedical and health informatics (2019).
Lee, Garam, et al. "Predicting Alzheimer's disease progression using multi-modal deep learning approach." Scientific reports 9.1 (2019): 1952.

Thank you for the comment and we totally agree. Misleading and unrelated references were removed. The proposed research (Spasov et al., Yang et al. Anf Garam et al.) have been added to the Related Work section.

4. It’s true ApoE information represents the most well-known genetic factor of AD, but having genetic data in the title while only using ApoE information is still misleading

The misleading title has been changed. We have replaced genetic with merely ApoE.

5. The left image in the Grad-CAM seems to pinpoint frontal lobe and makes sense, perhaps it’s better to visualize in 3D, see e.g. Feng, Xinyang, et al. "Deep Learning on MRI Affirms the Prominence of the Hippocampal Formation in Alzheimer's Disease Classification." bioRxiv (2018): 456277.

Thank you for the comment. The adopted approach is a 2D classification model and can unfortunately not be used to create similar Grad-CAM visualization as seen in Feng et al.

6. Using only clinical data (input #4) is not a good comparison

Thank you for the comment. The usage of merely clinical data has been completely removed from the manuscript. As Reviewer 1 mentioned, the term “clinical data” is misleading and was changed to “participants data”
