# Supplementary material for: Sociodemographic data and APOE-ε4 augmentation for MRI-based detection of amnestic mild cognitive impairment using deep learning systems
Source: PLoS One. 2020 Sep 25;15(9):e0236868. doi: 10.1371/journal.pone.0236868 (PMC7518632; doi:10.1371/journal.pone.0236868)
Supplement: Attachment — Submitted filename: Response_to_Reviewers.docx [file pone.0236868.s002.docx]

**Reviewer 3**

**1. Can you expand on the number of patients that were removed from each of the exclusion criteria’s?**

*Participants with dementia (n=7), severe depression (ADAS depression subscale score >4, n=13), Parkinson disease (n=5), mental retardation (n=2), severe alcohol consumption (for women: >20 g/day; for men: >40 g/day, n=2), known brain cancer (n=1), severe problems with the German language (foreign persons, n=9) and severe sensory impairment (n=2) leading to invalid cognitive testing were excluded from the sub-study.*

***2. Can you expand on why the ADNI dataset had lower performance despite having more data to train on.***

*In contrast to the ADNI data set the data in the HNR stem from a local cohort of German nationality  in three neighborhood cities in  the Ruhr Area. As a consequence this study population is rather homogenous both in cultural as in ethnic aspects. The HNR research group has consistently experienced similar obervations also in other fields, like CVD prediction.*

***3. On line 31-33 you state “However, a major concern in the medical domain is the lack of publicly available large image data sets like ImageNet [16]. This is due to the fact that detailed annotation of medical images is time-consuming, prone to errors and restricted by data protection rules” Minor point but can you just change it from lack of publicly available image data sets to insufficient number of datasets. They exist they’re just not enough for all contexts see.***

*Thank you for the comment. We have edited the statement and the required references (Wang et. al and Poldrack et. al) were added.*

***4. 89-90 “deep learning model, thereby exploring the potential of Transfer 89 Learning [15] from pre-trained ImageNet models” I would imagine using transfer learning from something from a medical domain like chestx-ray8 database would be better?***

*We adopted the chestx-ray8 database weights instead of imagenet to extract visual representations prior to training. This step was implemented for both the HNR study dataset, as well as the ADNI phase I dataset. The obtained evaluation metric scores are listed alongside, for comparison purposes.*

***5. Can you expand on the performance impact of the augmented 2 color layers?***

*Thank you for the comment. We have added information regarding the prediction rate improvements.*

***6. You use inception v3 as the basis of the transfer learning. Can you expand on why you chose that instead of a model pretrained for something in a medical context. As I mention in point 3 they exist.***

*We have included the prediction performance using the classification model with transfer learning using the chestx-ray8 database.*

***7. How does the model perform if you train it only on the HNR study dataset and then test it on the ADNI phase 1 dataset***

*Thank you for the comment. This experiment was performed and obtained less favourable prediction rates.*
